# Primary pulmonary mucoepidermoid carcinoma: a case report

**DOI:** 10.3389/fmed.2026.1893850

**Published:** 2026-10-12

**Authors:** Shaoyong Dong, Jing Sun, Ying Yang

**Affiliations:** 1 Department of Thoracic Surgery, Affiliated Hospital of Hebei University, Baoding, Hebei, China; 2 Department of Respiratory and Critical Care Medicine, Affiliated Hospital of Hebei University, Baoding, Hebei, China

**Keywords:** case report, CRTC1-MAML2, CRTC1-MAML2 gene fusion, low-grade, pulmonary mucoepidermoid carcinoma

## Abstract

Primary pulmonary mucoepidermoid carcinoma(PMEC) is a rare salivary gland-type malignancy, accounting for fewer than 0.2% of all primary lung tumors. We report herein a 61-year-old male patient with PMEC who was admitted for a space-occupying lesion in the left upper lobe and had a 20-year smoking history. Contrast-enhanced chest CT and positron emission tomography-computed tomography (PET-CT) revealed a central space-occupying lesion in the left hilum with distal obstructive inflammation. Flexible bronchoscopy revealed a nodular mass in the left main bronchus. Biopsy histopathology combined with immunohistochemistry confirmed the diagnosis of PMEC. Immunohistochemistry showed CK7 (+), P63 (-), P40 (-), CD117 (focal+), TTF-1 (-), NapsinA (-), and Ki-67 (7%+). Molecular testing identified a CRTC1-MAML2 gene fusion, whereas no classic driver gene mutations (e.g., EGFR or KRAS) were detected. PD-L1 expression was negative, and the tumor mutational burden (TMB) was low. The low Ki-67 index and positive MAML2 fusion in this case suggest a low-grade PMEC with a relatively favorable prognosis. The present study discusses the clinicopathological features, imaging findings, molecular diagnosis, and therapeutic advances of PMEC, along with a review of the relevant literature, aiming to increase clinical awareness of this rare disease.

## Introduction

Pulmonary mucoepidermoid carcinoma (PMEC) is a rare primary pulmonary salivary gland-type malignancy, accounting for only 0.1%–0.2% of all primary lung neoplasms. It is thought to arise from the submucosal glands of the tracheobronchial tree, predominantly affects middle-aged and elderly individuals, and has a slightly higher incidence in males than in females (1, 2). Owing to its low incidence and non-specific clinical manifestations, PMEC is prone to misdiagnosis. This study presents the clinicopathological data of a case of PMEC to enhance clinical awareness of this rare entity.

## Case report

A 61-year-old male patient presented to our hospital on March 1, 2026(Day 0), with a two-day history of intermittent dry cough and mild chest tightness. He had undergone a routine health check-up at a local hospital on February 28, 2026(Day -1), where a plain chest CT first revealed a space-occupying lesion in the left upper lobe, prompting his referral to our center. His past medical history was unremarkable for hypertension, diabetes, or tuberculosis; he had no prior surgeries or known malignancies. On admission, physical examination revealed decreased breath sounds in the left upper lung field, with no palpable lymphadenopathy or digital clubbing. His Eastern Cooperative Oncology Group (ECOG) performance status was 1. Laboratory findings were within normal limits, including complete blood count, liver and renal function tests, and tumor markers (CEA, CYFRA21-1, and NSE). Pulmonary function tests showed a forced expiratory volume in 1 s (FEV1) of 2.15 L (78% of predicted) and a ratio of FEV1 to forced vital capacity (FEV1/FVC) of 72%, indicating mild obstructive ventilatory impairment. He had a 20-year smoking history of approximately 20 cigarettes per day, with no significant occupational exposure.

Contrast-enhanced chest CT on March 5, 2026 (Day 4)(Figures 1,2) revealed a left hilar mass, a suspected malignancy, with distal obstructive inflammation and multiple bilateral pulmonary nodules. PET-CT further characterized the left hilar mass as hypermetabolic, suggestive of bronchogenic carcinoma, with mildly increased metabolism in the adjacent strip-like opacities consistent with inflammatory changes. Small mediastinal and bilateral hilar lymph nodes showed mild hypermetabolism, considered reactive. No hypermetabolic lesions suggestive of distant metastasis were identified.

**Figure 1:**
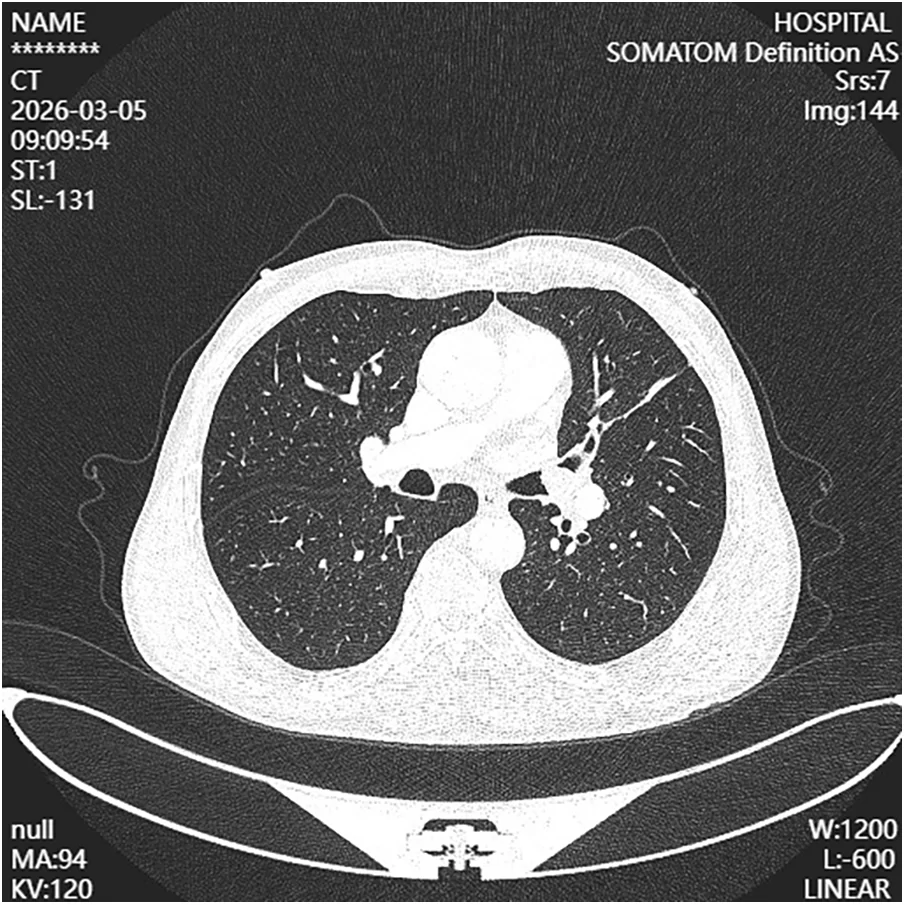
Contrast-enhanced chest CT shows a left hilar mass (arrow) with distal obstructive inflammation.

**Figure 2:**
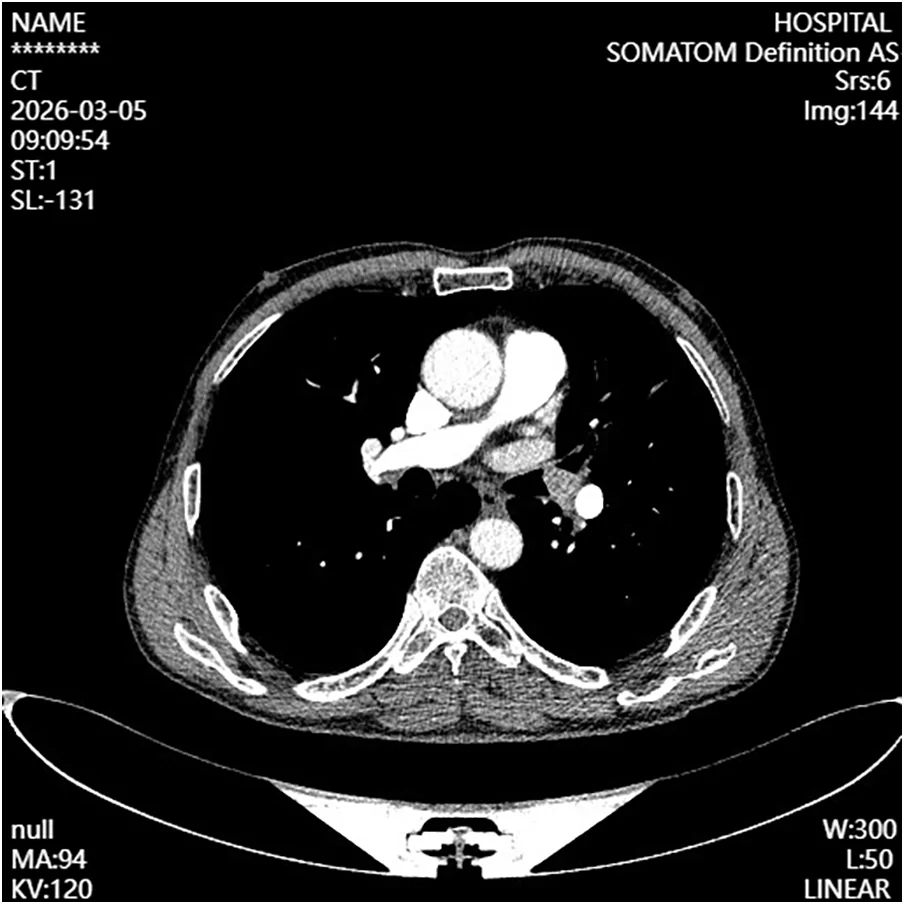
Contrast-enhanced chest CT shows a left hilar mass (arrow) with distal obstructive inflammation.

Flexible bronchoscopy on March 2, 2026 (Day 1) (Figure 3) revealed a nodular, friable endobronchial mass with abundant superficial vessels and a tendency to bleed upon contact. The tumor originated from the left secondary carina (the bifurcation between the left upper and lower lobe bronchi) and protruded proximally into the left main bronchus, occupying approximately 60%–70% of the luminal diameter at the level of the left main bronchus. The mass was partially obstructing but allowed passage of the bronchoscope distal to the lesion. Biopsy specimens were obtained from the mass for histopathological and molecular studies.

**Figure 3:**
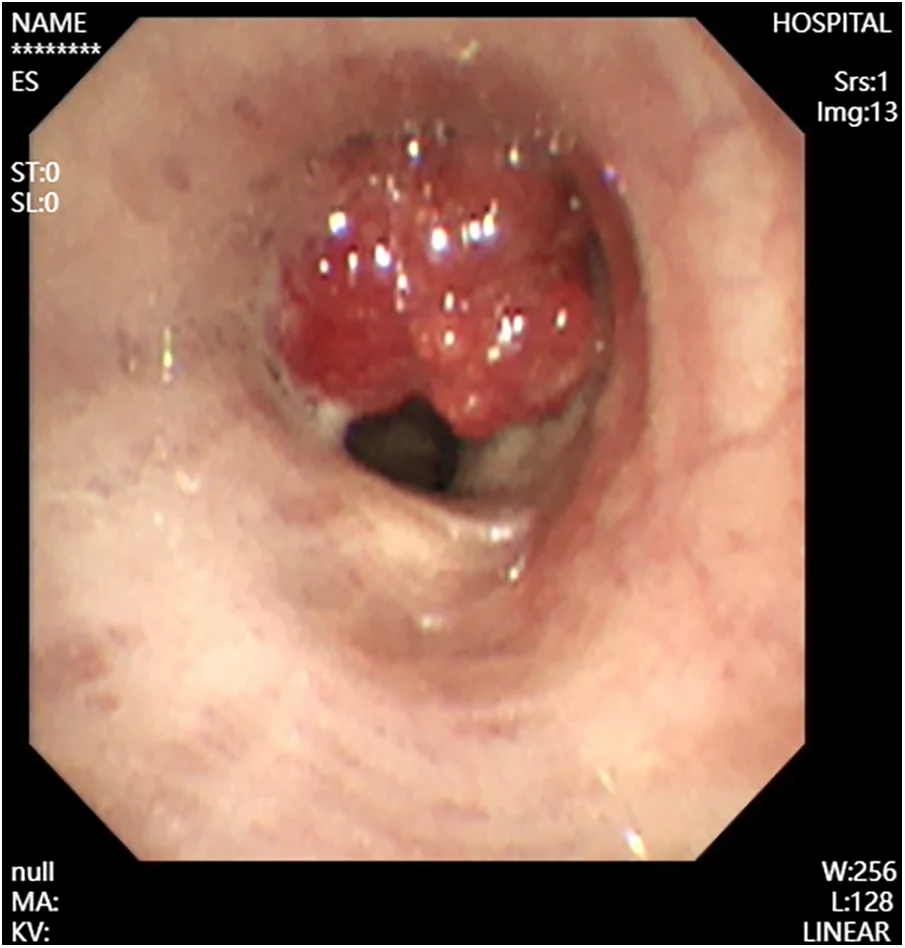
Flexible bronchoscopy on march 2, 2026.

Histopathological examination of the biopsy specimens revealed a tumor composed predominantly of nests and sheets of polygonal cells with moderate eosinophilic cytoplasm and mildly atypical nuclei, set within a fibrovascular stroma. Scattered interspersed mucous cells were identified, but the characteristic large mucinous glands typically seen in conventional mucoepidermoid carcinoma of the salivary gland were conspicuously sparse. The epidermoid component exhibited moderate nuclear atypia with occasional mitotic figures, whereas the intermediate cell population formed the predominant cellular constituency. No significant necrosis or perineural invasion was identified in the biopsy material (Figure 4). The overall morphological appearance was atypical for a classic PMEC, prompting the diagnostic team to consider a broad differential that included squamous cell carcinoma, poorly differentiated adenocarcinoma, and other salivary gland-type tumors such as adenoid cystic carcinoma and epithelial-myoepithelial carcinoma. Given this morphological ambiguity, an immunohistochemical panel was strategically employed to refine the differential diagnosis.

**Figure 4:**
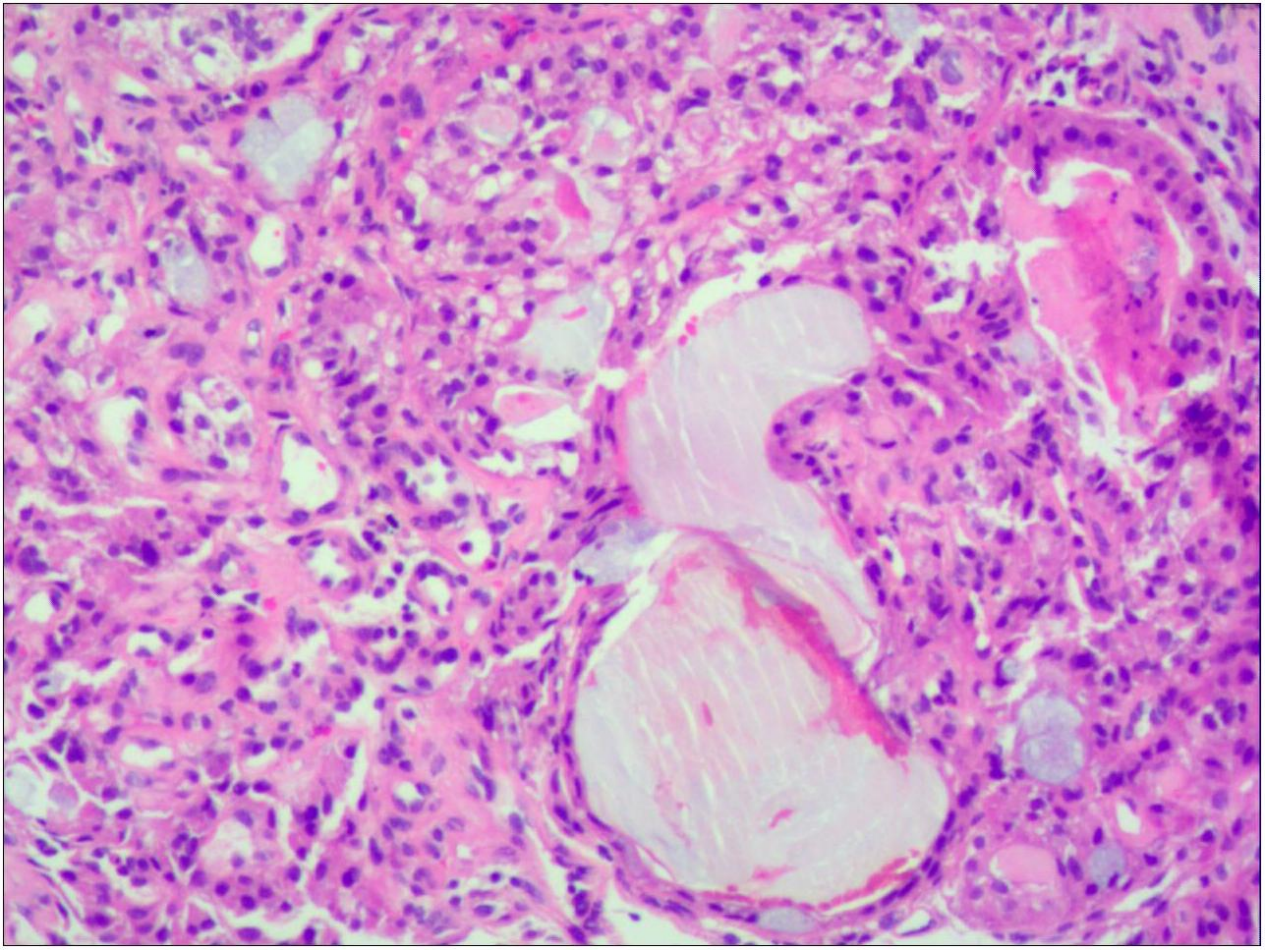
Subsequent pathological examination.

Molecular testing was performed on RNA extracted from the formalin-fixed paraffin-embedded (FFPE) biopsy specimens. CRTC1-MAML2 fusion transcript detection was carried out using targeted RNA sequencing with an anchored multiplex PCR-based enrichment panel (Archer® FusionPlex® Lung Panel, ArcherDx, Boulder, CO, USA), which employs anchored multiplex PCR to detect known and novel fusion events involving ALK, RET, ROS1, NTRK1/2/3, MET, and other relevant genes, including MAML2. Library preparation and sequencing were performed on the Illumina NextSeq 550 platform (Illumina, San Diego, CA, USA) with 2*150 bp paired-end reads. Sequence data were analyzed using the Archer Analysis software (version 6.0). A fusion was considered positive when supporting reads exceeded the predefined threshold of ≥5 unique fusion reads and the fusion junction spanned a breakpoint within the MAML2 exon 2 region, in accordance with the manufacturer's guidelines. This assay identified a CRTC1-MAML2 gene fusion (CRTC1 exon 2 fused to MAML2 exon 2), confirming the molecular hallmark of PMEC. No other targetable fusion variants (involving ALK, ROS1, RET, NTRK1/2/3, or MET) were detected. In parallel, comprehensive DNA sequencing was performed using a nex-generation sequencing panel (FoundationOne® CDx, Foundation Medicine, Cambridge, MA, USA) to assess for actionable driver mutations. No classic targetable driver mutations (EGFR, KRAS, BRAF, HER2, MET exon 14 skipping) were identified; only two missense variants of unknown significance (ABL2 and PPM1D) were detected. PD-L1 immunohistochemistry (22C3 pharmDx assay, Dako, Agilent, Santa Clara, CA, USA) showed TPS = 0% and CPS = 1, indicating negative PD-L1 expression. Tumor mutational burden (TMB) was calculated from the sequencing data and determined to be 2.0 mutations/Mb (low TMB), and microsatellite status was stable (MSS). All drug metabolism-related genes (DPYD, UGT1A1, CYP2D6) were wild-type, indicating no increased risk of chemotherapy toxicity. The complete timeline of diagnostic events, from initial detection to final pathological confirmation, is summarized in Table 1.

**Table 1:** Timeline of diagnostic events.

Date	Day	Event
February 28, 2026	Day -1	Initial CT
March 1, 2026	Day 0	Hospital admission
March 2, 2026	Day 1	Bronchoscopy
March 5, 2026	Day 4	Contrast-enhanced CT

## Discussion

This case of primary pulmonary mucoepidermoid carcinoma (PMEC) is noteworthy for several reasons. First, the diagnosis was established through a combination of flexible bronchoscopy, histopathology, and molecular testing, with the detection of a CRTC1-MAML2 gene fusion—a hallmark genetic alteration of PMEC—which not only confirmed the diagnosis but also supported a low-grade tumor with a favorable prognosis. Second, the comprehensive molecular profiling performed in this case, including PD-L1 testing, TMB assessment, and next-generation sequencing for actionable driver mutations, provided a complete picture of the tumor's molecular landscape, which is rarely reported in PMEC cases. Third, despite the patient's advanced age (61 years) and a 20-year smoking history, the tumor exhibited an indolent biological behavior (Ki-67 7%, negative PD-L1, low TMB), highlighting the importance of molecular characterization in distinguishing PMEC from more aggressive non-small cell lung cancers that share similar clinical and imaging features. This case underscores the value of integrating molecular diagnostics into the routine workup of rare pulmonary tumors and reinforces that low-grade PMEC carries an excellent prognosis after complete surgical resection, even in elderly patients with significant smoking exposure.

According to the 2021 WHO Classification of Thoracic Tumours (5th edition), PMEC is a rare salivary gland-type malignancy that is thought to arise from the seromucinous glands in the submucosa of the trachea and large bronchi. It is characterized by the absence of TTF-1 and Napsin A expression and frequently harbors MAML2 gene rearrangements (3). Histologically, the tumor is composed of a variable mixture of mucous cells, epidermoid (squamous) cells, and intermediate cells (4).

Central type PMEC, the most common, arises in the lobar, segmental, or more proximal bronchi; it predominantly involves the lobar bronchi, followed by the main bronchi, and is rare in smaller bronchi (5). PMEC lacks specific clinical manifestations, and symptoms are largely determined by tumor location and size. Small peripheral lesions are usually asymptomatic, whereas central space-occupying lesions frequently cause cough, hemoptysis, or post-obstructive inflammation due to airway invasion, which increases the diagnostic challenge (6). CT, contrast-enhanced CT, and PET-CT are effective tools for the initial evaluation of pulmonary masses. Regarding the CT imaging features of PMEC, Cheng DL et al. (7) reported that PMEC typically presents as a well-defined central endobronchial mass, often with distal obstructive pneumonia or atelectasis. Punctate calcification within the lesion is one of its characteristic findings, and the degree of tumor enhancement correlates with pathological grade. For differential diagnosis, Li X et al. (6) compared the imaging differences between PMEC and pulmonary adenoid cystic carcinoma (PACC) and found that PMEC tends to present as a polypoid intraluminal mass with lower non-contrast CT attenuation and less contrast enhancement than PACC. PACC, being rich in stroma, more often manifests as diffuse infiltration or longitudinally extending airway wall thickening. Collectively, the findings of central intraluminal nodules, punctate calcification, and mild-to-moderate enhancement on multi-detector CT, although not entirely specific, can help narrow the differential diagnosis and distinguish PMEC from other salivary gland-type tumors (e.g., adenoid cystic carcinoma), especially when combined with the clinical context of a relatively young age at onset (6, 7). In the present case, contrast-enhanced CT demonstrated several characteristic features consistent with PMEC: a well-defined central endobronchial mass at the left hilar region, with distal obstructive inflammation in the left upper lobe-findings that closely match the classic description of central-type PMEC. However, no definite punctate calcification was identified within the lesion, and the mass showed moderate enhancement on contrast imaging. These features, while suggestive of a salivary gland-type tumor, were not entirely specific. The absence of diffuse airway wall thickening or longitudinally extensive infiltration helped to exclude PACC, which typically presents with such findings (6). PET-CT further contributed to staging by demonstrating that the left hilar mass was the sole hypermetabolic lesion of concern, with no definitive evidence of distant metastasis; the small mediastinal and hilar lymph nodes showed only mild uptake, suggestive of reactive rather than metastatic involvement, which was later confirmed by the absence of nodal metastasis on pathological examination. Thus, imaging in this case played a crucial role in narrowing the differential diagnosis, guiding the decision to perform flexible bronchoscopy for tissue acquisition, and providing staging information that supported surgical planning. Given the low incidence and non-specific clinical and imaging features of PMEC, diagnosis still relies primarily on histopathology and immunohistochemistry. In the present case, flexible bronchoscopy was used to obtain tissue for pathological diagnosis.

Histological diagnosis of PMEC depends on the coexistence of three components: mucous cells, epidermoid cells, and intermediate cells. PMEC is graded into low-grade and high-grade based on cellular atypia, mitotic count, extent of necrosis, and invasiveness (8). Immunohistochemistry played a crucial role in navigating the diagnostic dilemma posed by the atypical morphology of this case. Given the morphological ambiguity-specifically, the paucity of large mucinous glands and the predominance of intermediate cells-a targeted immunohistochemical panel was employed to systematically exclude the differential diagnoses considered. The panel and its diagnostic implications were as follows. First, the absence of TTF-1 and Napsin A expression effectively ruled out pulmonary adenocarcinoma, despite the glandular appearance of some tumor cells (9, 10). Second, the negative staining for p63 and p40 argued against a diagnosis of primary pulmonary squamous cell carcinoma, which would be expected to show diffuse and strong positivity for these markers. Third, the focal positive staining for CD117, while observed in a subset of PMEC cases, raised the possibility of adenoid cystic carcinoma (ACC), a salivary gland-type tumor that frequently shows diffuse CD117 expression. However, the morphological absence of the characteristic cribriform or tubular architecture of ACC, together with the lack of perineural invasion, helped to exclude this diagnosis. Fourth, the positive CK7 staining confirmed the epithelial nature of the tumor but was non-specific, as CK7 is expressed in a wide range of carcinomas. Finally, the low Ki-67 proliferation index (7%) supported an indolent biological behavior, consistent with a low-grade neoplasm rather than a high-grade carcinoma (8). The immunophenotype—CK7 (+), TTF-1 (-), Napsin A (-), p63 (-), p40 (-), CD117 (focal +)—was unusual. P63 and p40 are expressed in the vast majority of PMECs. In the largest immunophenotypic series to date, Roden et al. (9) found p63 positivity in all 25 evaluable tumors and p40 positivity in 23 of 25 tumors (the two p40-negative tumors remained p63-positive), and Huo et al. (10) reported diffuse p63 and p40 positivity in all 26 cases studied. To the best of our knowledge, a p63/p40 double-negative PMEC has not been documented in these large series. The double-negative immunoprofile observed in the present case is therefore exceedingly rare and, in isolation, would argue against the diagnosis of PMEC. However, in the context of a pulmonary tumor with salivary gland-type morphology and after the exclusion of the main differential diagnoses (pulmonary adenocarcinoma, squamous cell carcinoma, and adenoid cystic carcinoma) by morphology and the remaining immunohistochemical panel, the possibility of PMEC could not be entirely dismissed on immunohistochemical grounds alone. The decisive diagnostic evidence came from molecular testing, which detected the pathognomonic CRTC1-MAML2 gene fusion, a hallmark genetic alteration present in the majority of PMEC cases (11, 12). This fusion confirmed the diagnosis of PMEC with certainty, demonstrating the essential complementary role of molecular diagnostics in cases where morphology and immunohistochemistry yield ambiguous results. In addition, the negative PD-L1 expression observed in this case is consistent with previous reports indicating that PD-L1 expression is rare in PMEC: in the largest immunohistochemical series of rare pulmonary tumors to date, tumor-cell PD-L1 positivity was documented in only 2 of 26 (7.7%) pulmonary mucoepidermoid carcinomas (13). Regarding TMB, systematic data in PMEC are scarce owing to the rarity of this entity; in mucoepidermoid carcinoma, high TMB has been reported in only a small minority of cases (approximately 15%), with the vast majority exhibiting low TMB (14), which is in line with the low TMB (2.0 mutations/Mb) observed in the present case. The low Ki-67 index (7%) further supported a low-grade PMEC with a relatively favorable prognosis (8).

Surgical resection remains the mainstay of treatment for PMEC. Complete (R0) resection combined with regional lymph node dissection is critical for improving long-term patient survival. For patients with low-grade, lymph node-negative, and margin-negative PMEC, the prognosis after surgery alone is favorable, with a 5-year survival rate exceeding 80%; adjuvant therapy is usually not required postoperatively (15). High-grade PMEC exhibits more aggressive biological behavior, with a high propensity for lymph node and distant metastasis. Even after radical resection, the risk of recurrence remains high, and the 5-year survival rate is significantly lower than that of low-grade PMEC (16). Large-scale retrospective studies based on the SEER database and nomogram prediction models have demonstrated that age, tumor size, lymph node status, and histological grade are independent prognostic factors for overall survival in PMEC patients (17). For patients with unresectable or recurrent/metastatic PMEC, radiotherapy and platinum-based chemotherapy remain the primary palliative options, albeit with limited overall efficacy (18). In recent years, prognostic nomogram models based on surgical strategies have also offered a reference for individualized treatment decision-making (19). Analysis of the present case: This 61-year-old male patient had a centrally located left hilar mass, and the pathological and molecular features (low Ki-67 index, positive MAML2 fusion) are suggestive of low-grade PMEC, which portends a relatively favorable prognosis. If subsequent complete (R0) resection with negative lymph nodes is achieved, the patient is expected to have long-term survival based on literature evidence, and postoperative adjuvant therapy may not be necessary.

Several strengths and limitations of this report should be acknowledged. The main strength lies in the comprehensive molecular characterization of the tumor, including the detection of the pathognomonic CRTC1-MAML2 gene fusion, PD-L1 testing, TMB assessment, and next-generation sequencing for actionable driver mutations. This level of molecular profiling is rarely reported in PMEC case studies and provides valuable reference data for the clinical management of this rare entity. In addition, the detailed documentation of the diagnostic timeline, from initial CT detection through bronchoscopic biopsy to final pathological confirmation, offers a practical roadmap for clinicians encountering similar cases. However, several limitations must be considered. First, this is a single case report, which limits the generalizability of our findings to the broader PMEC population. Second, the patient has not yet undergone surgical resection at the time of this report; therefore, long-term follow-up data and final pathological staging are not available to confirm the predicted low-grade nature and favorable prognosis. Third, the p63/p40 double-negative immunophenotype observed in this case is exceedingly rare and has not, to our knowledge, been reported in large PMEC cohorts; the diagnosis in this instance therefore rested critically on the molecular detection of the CRTC1-MAML2 fusion, and our immunohistochemical findings should be interpreted with this caveat in mind. Future studies with larger cohorts and longer follow-up are needed to further validate the prognostic implications of the molecular features observed in this case.

## Conclusion

In conclusion, PMEC is a rare primary pulmonary malignancy with non-specific clinical manifestations. Its diagnosis requires a comprehensive approach integrating imaging, histopathology, and molecular pathology. Complete surgical resection is the first-line treatment, and low-grade PMEC carries a favorable prognosis after standardized management. Clinicians should heighten awareness of this disease to avoid misdiagnosis; early diagnosis and standardized treatment are essential to improving patient outcomes.

## Data Availability

The original contributions presented in the study are included in the article/Supplementary Material, further inquiries can be directed to the corresponding author.
